# Cine interleaved sequences enabled imaging of mice on clinical 3T MRI and analysis of their cardiac function after myocardial infarction

**DOI:** 10.1186/1532-429X-14-S1-P60

**Published:** 2012-02-01

**Authors:** Alexandre Belin, Vincent Braunersreuther, Fabrizio Montecucco, Benedicte M Delattre, Francois Mach, Jean Vallee

**Affiliations:** 1 Radiology, HUG, Geneve 14, Switzerland; 2 Faculty of medicine, University of Geneva, Geneva, Switzerland

## Background

With the poor availability of small animal dedicated MRI, it is of great interest and challenge to use clinical MRI to image and analyze the cardiac function of mice. This would of course be advantageous for translational research and also enable the use of up-to-date sequences already implemented in clinical routine. The aim is to study the time evolution of cardiac function in mice with a myocardial infarct on a clinical MRI.

## Methods

C57BL/6 mice (n=4) were submitted in vivo to left coronary artery permanent ligature and compared and with compared with non-operated mice (n=4). The mice were imaged on a clinical Siemens 3T MRI using an “interleaved” sequence constructed from an ECG-triggered turboflash cine sequence, combining two acquisitions shifted in time yielding an effective time resolution of 6.8 ms and 20-26 phases per heart beat with following parameters: FOV 111 mm, in-plane resolution 257 μm, slice thickness 1 mm, TE/TR 6.2/13.5 ms, flip angle 30°. A soft-thresholding of the temporal Fourier coefficients was used to further denoise the images. The mice were scanned at 24h and 22 days after coronary ligation.

## Results

**Figure 1:**
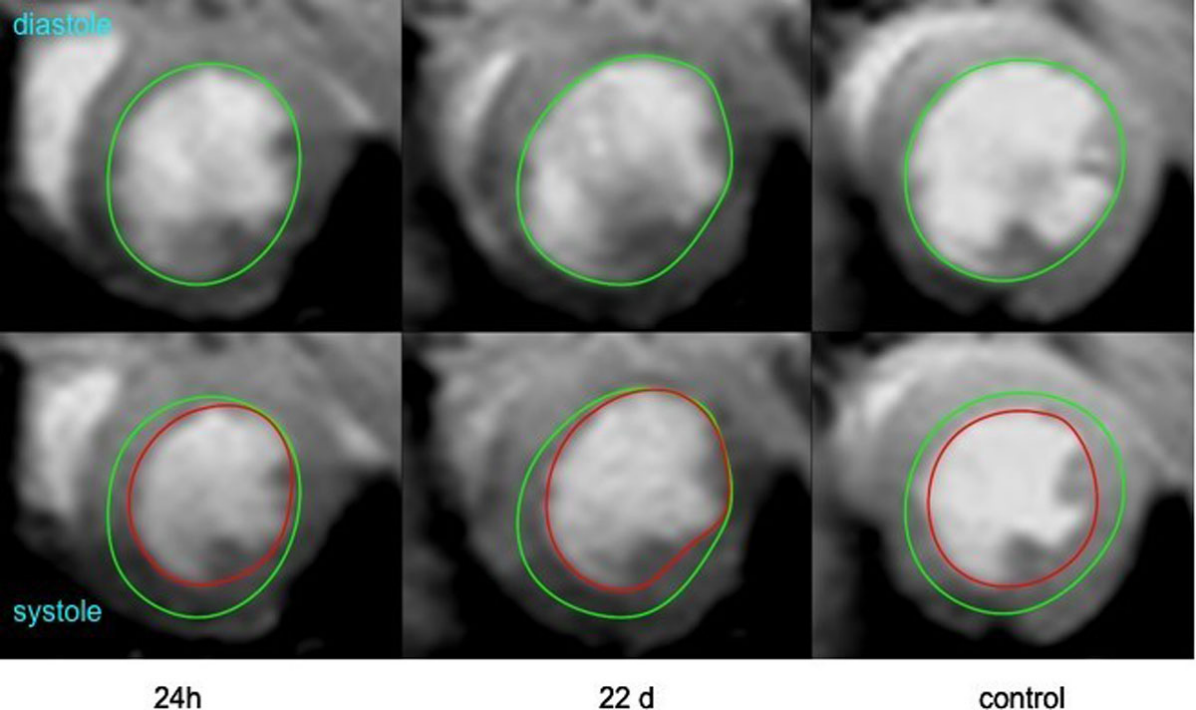


High quality images suitable for endocardial contouring were obtained for all the animals. The systolic and diastolic volumes, as well as the ejection fraction, are reported in Table 1. The anterior wall of all the operated animals was akenetic as shown on Figure 1. The ejection fraction was significantly decreased in the infarcted group at 24h and 22 days by comparison to the control group (p=0.006 and p=0.003). There was also a trend for the global function to decrease from 24h to 22 days.

**Table 1:** Cardiac properties of the mice

	ESV (ul)	EDV (ul)	EF
INF 24h			

EVAMR135	65.26	88.12	0.26
EVAMR90	51.27	69.73	0.26
EVAMR174	43.66	70.13	0.38
EVAMR175	51.27	81.90	0.37
MEAN	52.87	77.47	0.32
SD	9.01	9.07	0.07

INF 22j			

EVAMR135	289.57	356.57	0.19
EVAMR90	208.26	241.62	0.14
EVAMR174	61.75	91.41	0.32
EVAMR175	66.65	109.09	0.39
MEAN	156.56	199.67	0.26
SD	111.71	124.23	0.12

NON INF			

P1	21.13	56.86	0.63
P2	33.92	69.12	0.51
P3	36.83	79.46	0.54
P4	47.59	90.64	0.47
MEAN	34.87	74.02	0.54
SD	10.88	14.43	0.07

## Conclusions

We demonstrated a robust protocol to study cardiac functions in mice with a myocardial infarct using a clinical 3T MRI. This methodology has a strong potential to study the effect of treatment in rodents.

## Funding

This work was partially supported by the Swiss National Science Foundation (grant PP00P2-123438).

